# Supplementary material for: New genotype invasion of dengue virus serotype 1 drove massive outbreak in Guangzhou, China
Source: Parasit Vectors. 2021 Feb 27;14:126. doi: 10.1186/s13071-021-04631-7 (PMC7910771; doi:10.1186/s13071-021-04631-7)
Supplement: Supplementary file 1 — Additional file 1: Table S1. Specific primers for amplification and sequencing of dengue virus serotype 1. [file 13071_2021_4631_MOESM1_ESM.doc]

**Table S1.** Specific primers for amplification and sequencing of dengue virus serotype 1

| Primers code | Forward primer | Start | Reverse primer | Start |
| --- | --- | --- | --- | --- |
| DV.1_1 | ACAACCAACGGAAAAAGAC | 99 | CCTCTTTCCTGCTTGCTAAC | 495 |
| DV.1_2 | ATGAACAGGAGGAAAAGAT | 377 | GTGTCTCAGAGCCCAGGT | 826 |
| DV.1_3 | AACACCGACGAGACAAAC | 690 | TTGTGACCTCCGTCTTCA | 1088 |
| DV.1_4 | AGGAGCTACGTGGGTGGA | 982 | GCAGTTGTTCCATGTTCTG | 1419 |
| DV.1_5 | CGTGTGCTAAGTTTAAGTGT | 1278 | TGTGCATTGCTCCTTCTT | 1718 |
| DV.1_6 | TACCACTGCCTTGGACCT | 1581 | GACTGGTTTTTCTTTGTCAG | 2029 |
| DV.1_7 | AGACCCAGCATGGAACTG | 1875 | CAGCAGAATCCCTATTCCTA | 2323 |
| DV.1_8 | CATGGGACTTCGGTTCTA | 2190 | TTCCACACACACCCTCCT | 2588 |
| DV.1_9 | ACCAATGAAGTCCACACC | 2483 | CCATATGTTTGTCGTGAA | 2923 |
| DV.1_10 | CCAAAATCATAGGAGCAGAT | 2781 | GCCCTGCTGTTTGTGTGA | 3218 |
| DV.1_11 | GCCAAAATCCCACACTCT | 3094 | TGTCCACTTCTCCTGACC | 3494 |
| DV.1_12 | CGTTTCAAAGGAGAAGAC | 3383 | TCTACAGATGCCACCAGAC | 3819 |
| DV.1_13 | GGGGATGGGAACAACGTA | 3679 | CCCCAGATTTTGTTTTCTG | 4122 |
| DV.1_14 | TTCCCTTTATGCCTGTCC | 4007 | TCCTTTATCTTCATGGTTCC | 4392 |
| DV.1_15 | CCGATTTATCACTGGAGA | 4275 | AACTTGGTTCCAGTCTCTT | 4724 |
| DV.1_16 | GGGCAGGTCTCAAGTAGG | 4612 | ATGGCACTGACGTAGGTA | 5013 |
| DV.1_17 | GCGAAGTTGGAGCCATAG | 4881 | GACGCATAGTGAAAGTGG | 5327 |
| DV.1_18 | TCTTAGCTCCCACAAGAGTT | 5181 | TGATTTGATGCTTGGAACA | 5623 |
| DV.1_19 | GCAATGCAGTTATCCAAGA | 5505 | TCCAATTCTTCCTCTCCTC | 5908 |
| DV.1_20 | GAGCCGACAGGGTAATAGA | 5775 | CCATCAAAGCACCACCTT | 6219 |
| DV.1_21 | GCAATAGACGGGGAATACA | 6074 | GTTCTTCCATGGCGTGTC | 6518 |
| DV.1_22 | AGGGAAACTTCCACAACA | 6406 | AGACCTATCACCACGTATGC | 6798 |
| DV.1_23 | GGAGTTCTTTCTGATGGTGT | 6709 | AGGCGAGAAGTGGAACTC | 7115 |
| DV.1_24 | GAAAACACAACGGCAAAT | 6998 | TCCAGTGGCTAGTGTGATG | 7426 |
| DV.1_25 | CGTTGCAATAGATTTGGAC | 7279 | CGTTTCTCCTCTTTTTAACC | 7723 |
| DV.1_26 | AGCCCAAGGGGAAACACT | 7582 | GGACTCACCAATATCACACA | 8023 |
| DV.1_27 | GGATACACGAAAGGAGGA | 7877 | ATGTTGGCTTCCTGTGAG | 8324 |
| DV.1_28 | GGAATGCTAGTGCGAAATC | 8177 | CAAAGGGTGTGGTGTCAG | 8618 |
| DV.1_29 | ATGAGGTCAAGCCATCAG | 8496 | TGAAGCTCCCTCTCTCTGT | 8895 |
| DV.1_30 | GCAGTGTTCGTTGATGAAA | 8801 | CCTCTGTTATTCTTGTGTCC | 9203 |
| DV.1_31 | CAGTGGAGTGGAAGGAGA | 9082 | TCTCGGTGCCATGTTTTT | 9530 |
| DV.1_32 | CATGGAGGCCCAACTAAT | 9412 | GCGCCTTGTGATACTCTG | 9801 |
| DV.1_33 | GGCAACAAGTGCCTTTCT | 9678 | AGGGAACCACACCATTGA | 10119 |
| DV.1_34 | TGGAATAGGGTTTGGATAGA | 10007 | CGGCCTGACTTCATTTTA | 10406 |
| DV.1_35 | AAGGGGCACTCTGGTAAG | 10257 | GTCTCTCCCAGCGTCAAT | 10653 |
