# Supplementary material for: New genotype invasion of dengue virus serotype 1 drove massive outbreak in Guangzhou, China
Source: Parasit Vectors. 2021 Feb 27;14:126. doi: 10.1186/s13071-021-04631-7 (PMC7910771; doi:10.1186/s13071-021-04631-7)
Supplement: Supplementary file 2 — Additional file 2: Table S2. Distribution of DENV-1 genome sequences in different countries and regions. [file 13071_2021_4631_MOESM2_ESM.doc]

**Table S2.** Distribution of DENV-1 genome sequences in different countries and regions

| **Country** | **Number of sequences** | **Proportion (%)** |
| --- | --- | --- |
| Viet Nam | 818 | 48.72 |
| Singapore | 124 | 7.39 |
| Mexico | 124 | 7.39 |
| **China** | **97** | **5.78** |
| Cambodia | 92 | 5.48 |
| Venezuela | 71 | 4.23 |
| Nicaragua | 66 | 3.93 |
| Puerto Rico | 48 | 2.86 |
| Indonesia | 36 | 2.14 |
| Thailand | 35 | 2.08 |
| Brazil | 33 | 1.97 |
| Argentina | 23 | 1.37 |
| Colombia | 17 | 1.01 |
| Myanmar | 15 | 0.89 |
| India | 13 | 0.77 |
| Sri Lanka | 11 | 0.66 |
| USA | 10 | 0.60 |
| French Polynesia | 8 | 0.48 |
| Japan | 6 | 0.36 |
| Malaysia | 4 | 0.24 |
| New Caledonia | 4 | 0.24 |
| Reunion | 3 | 0.18 |
| South Korea | 3 | 0.18 |
| Laos | 3 | 0.18 |
| Philippines | 2 | 0.12 |
| Brunei | 2 | 0.12 |
| Nauru | 1 | 0.06 |
| France | 1 | 0.06 |
| Haiti | 1 | 0.06 |
| Angola | 1 | 0.06 |
| El Salvador | 1 | 0.06 |
| British Virgin Islands | 1 | 0.06 |
| Chile | 1 | 0.06 |
| Saudi Arabia | 1 | 0.06 |
| Germany | 1 | 0.06 |
| Seychelles | 1 | 0.06 |
| Comoros | 1 | 0.06 |
| **Total** | **1679** | **100.00** |
