# Supplementary figures and images for: New genotype invasion of dengue virus serotype 1 drove massive outbreak in Guangzhou, China
Source: Parasit Vectors. 2021 Feb 27;14:126. doi: 10.1186/s13071-021-04631-7 (PMC7910771; doi:10.1186/s13071-021-04631-7)

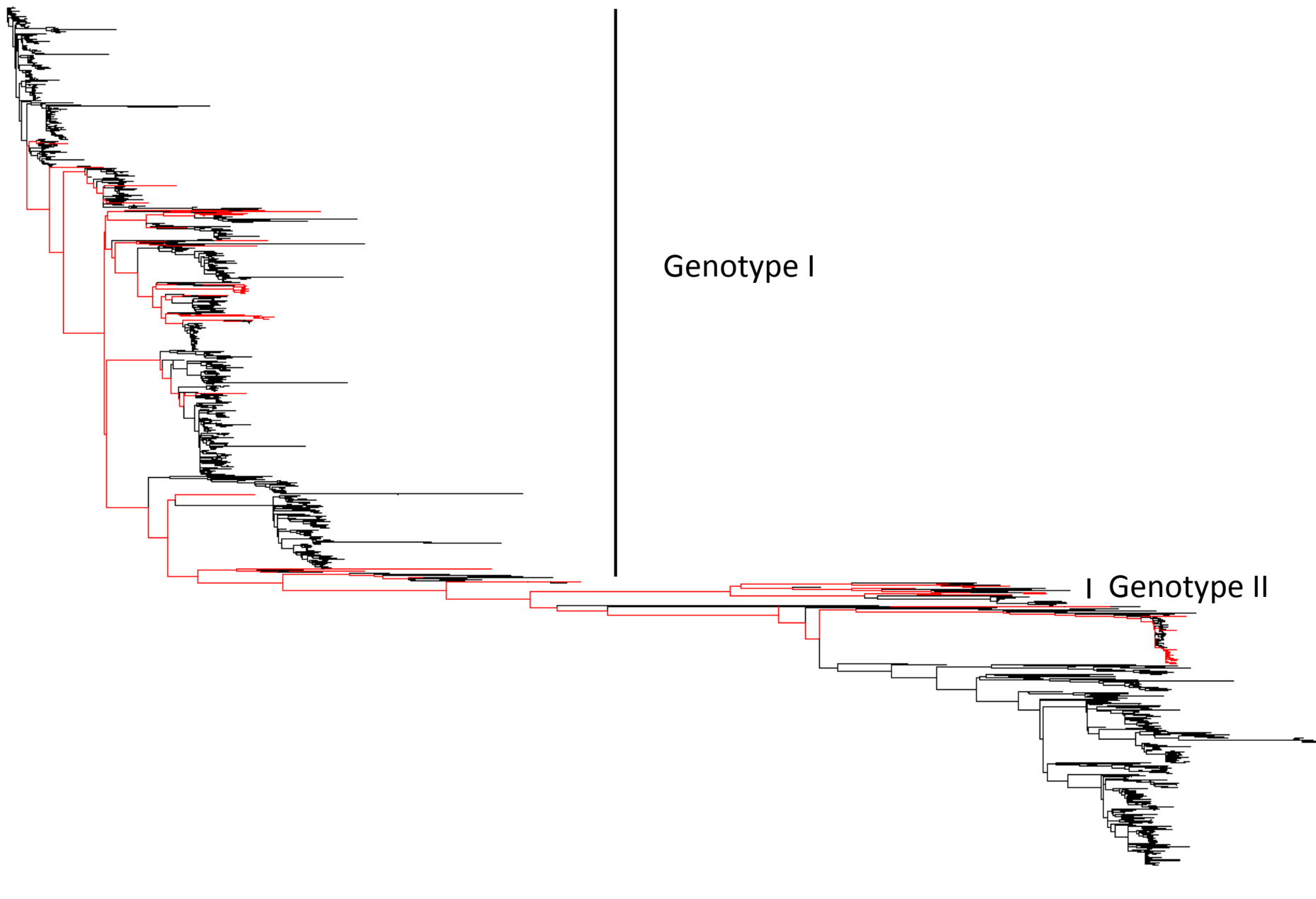

Genotype I

Genotype II

Genotype III

Supplement: Supplementary file 3 — Additional file 3: Fig S1. DENV-1 complete genome sequence phylogenetic tree. [file 13071_2021_4631_MOESM3_ESM.pdf]
