# Supplementary material for: New genotype invasion of dengue virus serotype 1 drove massive outbreak in Guangzhou, China
Source: Parasit Vectors. 2021 Feb 27;14:126. doi: 10.1186/s13071-021-04631-7 (PMC7910771; doi:10.1186/s13071-021-04631-7)
Supplement: Supplementary file 4 — Additional file 4: Fig S2. Phylogenetic trees for each protein and complete coding sequence of DENV-1 genotype III in Guangzhou (UPGMA method). [file 13071_2021_4631_MOESM4_ESM.pdf]

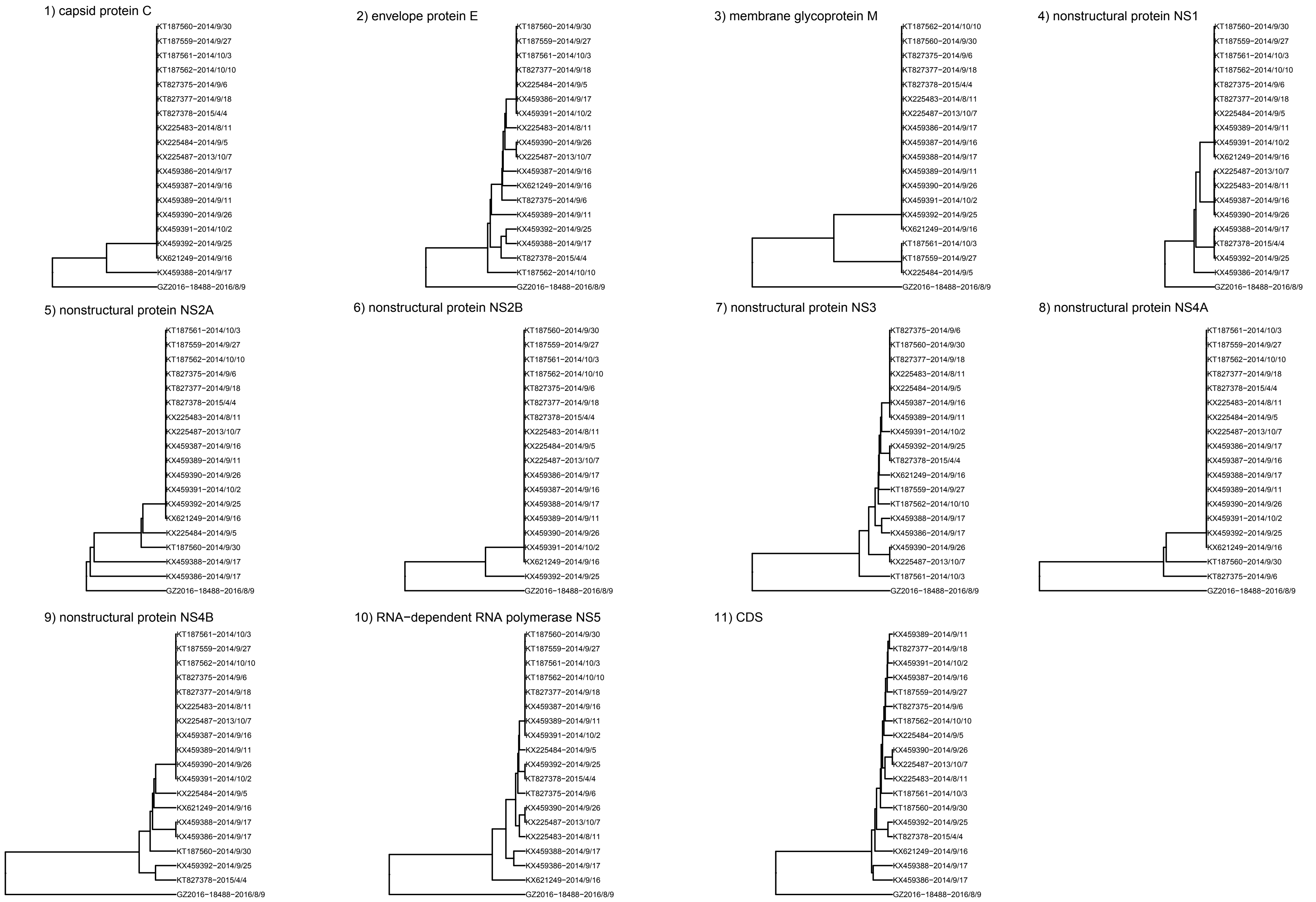
1) capsid protein C
KT187560−2014/9/30
KT187559−2014/9/27
KT187561−2014/10/3
KT187562−2014/10/10
KT827375−2014/9/6
KT827377−2014/9/18
KT827378−2015/4/4
KX225483−2014/8/11
KX225484−2014/9/5
KX225487−2013/10/7
KX459386−2014/9/17
KX459387−2014/9/16
KX459389−2014/9/11
KX459390−2014/9/26
KX459391−2014/10/2
KX459392−2014/9/25
KX621249−2014/9/16
KX459388−2014/9/17
GZ2016−18488−2016/8/9
2) envelope protein E
KT187560−2014/9/30
KT187559−2014/9/27
KT187561−2014/10/3
KT827377−2014/9/18
KX225484−2014/9/5
KX459386−2014/9/17
KX459391−2014/10/2
KX225483−2014/8/11
KX459390−2014/9/26
KX225487−2013/10/7
KX459387−2014/9/16
KX621249−2014/9/16
KT827375−2014/9/6
KX459389−2014/9/11
KX459392−2014/9/25
KX459388−2014/9/17
KT827378−2015/4/4
KT187562−2014/10/10
GZ2016−18488−2016/8/9
3) membrane glycoprotein M
KT187562−2014/10/10
KT187560−2014/9/30
KT827375−2014/9/6
KT827377−2014/9/18
KT827378−2015/4/4
KX225483−2014/8/11
KX225487−2013/10/7
KX459386−2014/9/17
KX459387−2014/9/16
KX459388−2014/9/17
KX459389−2014/9/11
KX459390−2014/9/26
KX459391−2014/10/2
KX459392−2014/9/25
KX621249−2014/9/16
KT187561−2014/10/3
KT187559−2014/9/27
KX225484−2014/9/5
GZ2016−18488−2016/8/9
4) nonstructural protein NS1
KT187560−2014/9/30
KT187559−2014/9/27
KT187561−2014/10/3
KT187562−2014/10/10
KT827375−2014/9/6
KT827377−2014/9/18
KX225484−2014/9/5
KX459389−2014/9/11
KX459391−2014/10/2
KX621249−2014/9/16
KX225487−2013/10/7
KX225483−2014/8/11
KX459387−2014/9/16
KX459390−2014/9/26
KX459388−2014/9/17
KT827378−2015/4/4
KX459392−2014/9/25
KX459386−2014/9/17
GZ2016−18488−2016/8/9
5) nonstructural protein NS2A
KT187561−2014/10/3
KT187559−2014/9/27
KT187562−2014/10/10
KT827375−2014/9/6
KT827377−2014/9/18
KT827378−2015/4/4
KX225483−2014/8/11
KX225487−2013/10/7
KX459387−2014/9/16
KX459389−2014/9/11
KX459390−2014/9/26
KX459391−2014/10/2
KX459392−2014/9/25
KX621249−2014/9/16
KX225484−2014/9/5
KT187560−2014/9/30
KX459388−2014/9/17
KX459386−2014/9/17
GZ2016−18488−2016/8/9
6) nonstructural protein NS2B
KT187560−2014/9/30
KT187559−2014/9/27
KT187561−2014/10/3
KT187562−2014/10/10
KT827375−2014/9/6
KT827377−2014/9/18
KT827378−2015/4/4
KX225483−2014/8/11
KX225484−2014/9/5
KX225487−2013/10/7
KX459386−2014/9/17
KX459387−2014/9/16
KX459388−2014/9/17
KX459389−2014/9/11
KX459390−2014/9/26
KX459391−2014/10/2
KX621249−2014/9/16
KX459392−2014/9/25
GZ2016−18488−2016/8/9
7) nonstructural protein NS3
KT827375−2014/9/6
KT187560−2014/9/30
KT827377−2014/9/18
KX225483−2014/8/11
KX225484−2014/9/5
KX459387−2014/9/16
KX459389−2014/9/11
KX459391−2014/10/2
KX459392−2014/9/25
KT827378−2015/4/4
KX621249−2014/9/16
KT187559−2014/9/27
KT187562−2014/10/10
KX459388−2014/9/17
KX459386−2014/9/17
KX459390−2014/9/26
KX225487−2013/10/7
KT187561−2014/10/3
GZ2016−18488−2016/8/9
8) nonstructural protein NS4A
KT187561−2014/10/3
KT187559−2014/9/27
KT187562−2014/10/10
KT827377−2014/9/18
KT827378−2015/4/4
KX225483−2014/8/11
KX225484−2014/9/5
KX225487−2013/10/7
KX459386−2014/9/17
KX459387−2014/9/16
KX459388−2014/9/17
KX459389−2014/9/11
KX459390−2014/9/26
KX459391−2014/10/2
KX459392−2014/9/25
KX621249−2014/9/16
KT187560−2014/9/30
KT827375−2014/9/6
GZ2016−18488−2016/8/9
9) nonstructural protein NS4B
KT187561−2014/10/3
KT187559−2014/9/27
KT187562−2014/10/10
KT827375−2014/9/6
KT827377−2014/9/18
KX225483−2014/8/11
KX225487−2013/10/7
KX459387−2014/9/16
KX459389−2014/9/11
KX459390−2014/9/26
KX459391−2014/10/2
KX225484−2014/9/5
KX621249−2014/9/16
KX459388−2014/9/17
KX459386−2014/9/17
KT187560−2014/9/30
KX459392−2014/9/25
KT827378−2015/4/4
GZ2016−18488−2016/8/9
10) RNA−dependent RNA polymerase NS5
KT187560−2014/9/30
KT187559−2014/9/27
KT187561−2014/10/3
KT187562−2014/10/10
KT827377−2014/9/18
KX459387−2014/9/16
KX459389−2014/9/11
KX459391−2014/10/2
KX225484−2014/9/5
KX459392−2014/9/25
KT827378−2015/4/4
KT827375−2014/9/6
KX459390−2014/9/26
KX225487−2013/10/7
KX225483−2014/8/11
KX459388−2014/9/17
KX459386−2014/9/17
KX621249−2014/9/16
GZ2016−18488−2016/8/9
11) CDS
KX459389−2014/9/11
KT827377−2014/9/18
KX459391−2014/10/2
KX459387−2014/9/16
KT187559−2014/9/27
KT827375−2014/9/6
KT187562−2014/10/10
KX225484−2014/9/5
KX459390−2014/9/26
KX225487−2013/10/7
KX225483−2014/8/11
KT187561−2014/10/3
KT187560−2014/9/30
KX459392−2014/9/25
KT827378−2015/4/4
KX621249−2014/9/16
KX459388−2014/9/17
KX459386−2014/9/17
GZ2016−18488−2016/8/9
